# Associations of socioeconomic status and lifestyle factors with dental neglect of elementary school children: the MEXT Super Shokuiku School Project

**DOI:** 10.1186/s12199-020-00916-y

**Published:** 2020-11-25

**Authors:** Yukiko Asaka, Michikazu Sekine, Masaaki Yamada, Takashi Tatsuse

**Affiliations:** 1 Department of Epidemiology and Health Policy, University of Toyama, 2630 Sugitani, Toyama, 930-0194 Japan; 2 Division of Pediatric Dentistry, Department of Human Development and Fostering, Meikai University School of Dentistry, 1-1 Keyakidai, Saitama, 350-0283 Japan

**Keywords:** Dental neglect, Socioeconomic status, Lifestyle, The Super Shokuiku School Project

## Abstract

**Abstract:**

**Background:**

Despite the fact that there are parents who do not take children with untreated dental caries to a dental clinic, few studies have been conducted to identify the responsible underlying social and family factors. The aim of this study was to investigate whether socioeconomic status and lifestyle factors are associated with dental neglect in elementary school children.

**Methods:**

This study was conducted in 2016 with 1655 children from the Super Shokuiku School Project in Toyama. Using Breslow’s seven health behaviors, the survey assessed: the grade, sex, and lifestyle of the children; parental internet and game use and lifestyle; socioeconomic status. The odds ratios (OR) and 95% confidence intervals (CIs) for having untreated dental caries were calculated using logistic regression analysis.

**Results:**

Among the children participating, 152 (3.2%) had untreated dental caries. Among them, 53 (34.9%) had not been taken to a dental clinic despite the school dentist’s advice. Dental neglect was significantly associated with children in higher grades (OR, 2.08; 95% CI, 1.14–3.78), father’s Internet and game use ≥ 2 h/day (OR, 1.99; 95% CI, 1.02–3.88), not being affluent (OR, 2.78; 95% CI, 1.14–6.81), and non-engagement in afterschool activities (OR, 1.99; 95% CI, 1.10–3.62).

**Conclusions:**

Socioeconomic status was the strongest factor associated with dental neglect despite the fact that the children’s medical expenses are paid in full by the National Health Insurance in Toyama, Japan. Future studies should investigate what factors prevent parents of non-affluent families from taking their children to dental clinics and how they can be socially supported to access adequate medical care.

## Background

Neglect of dental treatment is becoming a serious healthcare problem. According to the School Dental Treatment Survey 2018, Japan, 52.1% of elementary school children whose parents were requested to have their children treated for dental caries by the school dentist were left untreated [1]. The American Academy of Pediatric Dentistry defined dental neglect as “willful failure of parent or guardian, despite adequate access to care, to seek and follow through with treatment necessary to ensure a level of oral health essential for adequate daily functioning and freedom from pain and infection.” [2]. This definition also includes parents who have been informed by a dentist that children have dental caries left untreated. Therefore, in order to determine dental neglect, it is important to consider whether parents are aware of their children’s need for treatment.

Untreated dental caries may cause pain, lack of sleep, low academic performance, and low engagement in social activity [2–5]. They are often accompanied by periodontal diseases [2]. In addition, severe deciduous caries may cause Turner’s teeth, in which the apical periodontitis of deciduous teeth may induce enamel hypoplasia in succeeding permanent teeth [5]. Moreover, it is widely accepted that a higher prevalence of dental caries in the permanent dentition is found when the deciduous teeth are highly affected by dental caries [5]. Furthermore, malnutrition due to severe caries inhibits normal growth and development [2–5]. Therefore, dental caries in children should be treated in a timely manner.

In this context, dental caries left untreated have been associated with socioeconomic status (SES), showing high incidence and prevalence rates among disadvantaged population groups [5–7]. Additionally, the lack of treatment of child dental caries is more prevalent among ethnic minority groups, those with low educational attainment, those living in rural areas, those with parental history of caries and poor dental hygiene habits, and those who skip breakfast [8, 9].

In Japan, the National Insurance System covers almost all medical expenses for dental caries, and only a very minimal payment is required to consult a dentist [10]. In some areas in Japan, children’s medical expenses are paid in full by the National Insurance System with the help of local government subsidiaries. Therefore, besides economic factors, there may be other factors that play a role in dental neglect.

However, few studies have examined these possible underlying factors comprehensively for children with dental neglect. The purpose of this study is, therefore, to identify the underlying social and family factors for children with dental neglect.

## Methods

### Participants

The Super Shokuiku School Project is a food education project funded by the Ministry of Education, Culture, Sports, Science and Technology (MEXT), aimed at maintaining desirable lifestyles and the physical and mental health of school children through food education for children and their parents. Detailed information and some of the results of The Super Shokuiku School Project have been published elsewhere [11–14].

A questionnaire survey was conducted in March 2016 with the children in the aforementioned project. Questionnaires were distributed to 2109 children from five elementary schools in Takaoka City, Toyama Prefecture, out of which, 1987 children responded (response rate, 94.2%), and among them, 1655 (817 boys and 838 girls) who answered all the questions relevant to this study were included in the analysis.

In Takaoka City, there are 16 semi-rural areas with a population of approximately 170,000 people. In this area, there are a higher number of dual-income households (53.9%) than the national average (45.4%), leading to household incomes that are also higher (55,686 USD) than the national average (50,163 USD) [15–18].

The Ethics Committee of the University of Toyama approved this study. Written informed consent was obtained from the respondents and their parents. All subjects participated in the study voluntarily.

### Questionnaires

An anonymous self-report questionnaire surveilling dietary habits and dietary education was distributed, and children answered the questionnaire under parental supervision. The questionnaires for the children collected information on sex, school grade (from 1 to 6), and lifestyle factors including breakfast habits, dinner habits, and dental caries. Questionnaires for parents included assessment of their own lifestyles, time spent on internet and game usage at home on a weekday, perceived family affluence, parental employment status, and the number of afterschool activities their children engaged in.

### Children’s lifestyle factors

Children reported their sex, grade, and dietary habits by choosing the most appropriate answer from among several options. Responses to the questions on school grade, breakfast habits, and on eating alone at dinner were each divided into two groups respectively, as follows: “lower grades of 1–3” and “higher grades of 4–6”; “having breakfast every day” or “sometimes to usually skipping breakfast”; “alone” or “with someone.”

“Skipping breakfast” and “eating alone at dinner” have been used as the poverty index [19, 20], while neglect is considered one of the most severe forms of maltreatment related to poverty [21]. Therefore, we used these indices in this study.

### Dental caries

Parents were requested to provide the details of their children’s oral health conditions, which were assessed in January 2016 by trained school dentists. The health screening is performed on a tri-annual basis in April, September, and January according to the School Health Law. School dentists shared the results of children’s dental examination and advised parents to have checkups at a dental clinic if students had dental problems (cavities, gingivitis, and so on). Parents were also instructed to send a diagnosis report to the home room teacher after the check-up. Responses to the number of treated dental caries were categorized into two groups: “< 3 treated teeth” or “≥ 3 treated teeth.” In the 2016 National Survey of Dental Diseases, the mean number of teeth with dental caries (i.e., total number of decayed, filled, or primary teeth [dft index]) ranged from 0.2 to 2.4 among elementary school children aged 6 to 12 [22]. Furthermore, the Japan Association of School Dentists declared that children aged 5 to 7 with ≥ 3 treated deciduous teeth or ≥ 1 treated permanent teeth are defined as a high-risk group. Meanwhile, children aged 8 to 10 with ≥ 2 treated permanent teeth and those aged 11 to 12 with ≥ 3 treated permanent teeth are defined as a high-risk group [23]. Using this as a reference, we defined ≥ 3 treated teeth as a “high-risk group.”

Participants were also asked to answer whether dental caries were pointed out by a school dentist during the dental checkup in the previous year and whether children with untreated dental caries have since seen a dentist. In this context, “dental neglect” was defined as those who had been informed of dental caries in the previous year but had not yet seen a dentist as of the time of survey [2].

A previous study showed that mothers’ perceptions (self-report) of the oral health of their children aged 6–13 years were associated with their children’s dental caries (OR, 2.54; 95% CI, 1.54–4.30) [24]. Further, another study demonstrated that mothers’ perceptions of the number of their children’s dental caries were associated with the number of dental caries as diagnosed by a dentist [25]. Therefore, the answer to the question of the number of dental caries is worthy of analysis.

### Parental lifestyle factors

Lifestyle factors were assessed by Breslow’s seven Health Practice Score (BHPS) [26], which measures respondents’ adherence to seven good health habits that are widely accepted in developed countries [14, 19]: (1) never smoking cigarettes, (2) engaging in regular physical activity, (3) drinking alcohol in moderation or not at all, (4) getting 7 to 8 h of sleep regularly, (5) maintaining proper weight, (6) eating breakfast, and (7) not eating between meals. The parents in this study gave yes/no answers to these seven items. The higher the number of healthy behaviors, the lower the incidence of lifestyle-related diseases. “Yes” responses were summed to provide a cumulative BHPS ranging from 0 to 7, which we categorized into two groups: poor (0–1) and not poor (2–7). Moreover, a previous study showed that parental lifestyle factors are associated with health problems in children [13, 14]. Hence, we used the BHPS in this research.

Parental internet and game usage time at home on weekdays was coded into two groups, “< 2 h” or “≥ 2 h”, based on the report that the average time spent on the internet by Japanese adults in their thirties was about 1.92 h in 2016 [27]. We have considered adding BHPS to internet usage time, resulting in health indicators that are more modern in this research.

### SES

Regarding SES, we asked parents about their perceived family affluence. Family affluence was divided into three groups: “affluent,” “neither,” and “not affluent.” Meanwhile, mother’s employment status was categorized into two groups: “full-time” and “other” (part time or unemployed). Since the number of part-time and unemployed fathers was too small, the variable of father’s employment status was not included in the analysis. The responses to the question about afterschool activities were categorized into two groups: “= 0” or “≥ 1” based on a report that these activities are associated with poverty outcomes [28–30].

### Statistical analysis

Descriptive statistics for the participants in this study were calculated. Chi-square tests were used to evaluate whether children with dental caries left untreated had different SES and lifestyles (both child and parental lifestyles) from those with no, treated, or being treated dental caries. Further, logistic regression analysis was used to evaluate the associations between untreated dental caries and family affluence, lifestyle factors of the parent, and lifestyle factors of the child. Next, the Hosmer-Lemeshow test was used to validate the multivariate model [31]. Statistical analyses were performed using SPSS version 20.0 (SPSS Japan Inc., Tokyo, Japan). *P* < 0.05 was accepted as statistically significant.

## Results

Table 1 shows the characteristics of the participants. Out of the 1987 who returned their questionnaires, 1655 children (817 boy and 838 girls) who answered all questions relevant to the purpose of this study were included in the analysis. In total, 71.6% (*n* = 1185) of children had caries (treated and untreated), and 9.18% (*n* = 152) had untreated dental caries. Overall, 3.2% (*n* = 53) children experienced dental neglect (i.e., they had dental caries left untreated despite it being recommended that they see a dentist the previous year). In the Pearson’s chi-square test, correlating factors for dental neglect were: being 4th–6th graders, number of treated dental caries ≥ 3, father’s poor BHPS (0–1), father’s Internet and game usage time ≥ 2 h/day, no family affluence, and no afterschool activities.

**Table 1:** Characteristics of the participants (*n* = 1655)

		Number and percentage of children with dental neglect			
		Total	*n*	%	*P* value
Children factors					
Sex	Boys	817	27	3.3	
	Girls	838	26	3.1	0.46
Grade	Low (1st–3rd)	829	17	2.1	
	High (4th–6th)	826	36	4.4	0.01
Skipping breakfast	No	1547	46	3.0	
	Yes	108	7	6.5	0.05
Eating alone at dinner	No	1635	51	3.1	
	Yes	20	2	10.0	0.13
Number of treated teeth	≤ 2	1021	25	2.4	
	≥ 3	634	28	4.4	0.02
Parental factors					
Father’s BHPS	Not poor (2–7)	1527	47	3.0	
	Poor (0–1)	77	6	7.2	0.05
Mother’s BHPS	Not poor (2–7)	1583	51	3.1	
	Poor (0–1)	19	2	9.5	0.14
Father’s Internet and game use at home, h/day	< 2 h	1416	39	2.8	0.02
	≥ 2 h	239	14	5.9	
Mother’s Internet and game use at home, h/day	< 2 h	1566	49	3.1	
	≥ 2 h	89	4	4.5	0.32
Social factors					
Perceived family affluence	Affluent	447	7	1.6	
	Neither	794	26	3.3	
	No	414	20	4.8	0.03
Mother’s employment status	Full-time	672	22	3.3	
	Part-time	742	19	2.6	
	Unemployment	241	12	5.0	0.18
Number of after school activities	≥1	1330	33	2.5	
	0	325	20	6.2	0.001

*BHPS* Breslow’s health practice score. *P* values in Pearson’s chi-square test were shown

Table 2 shows the association between child and parental factors and dental neglect. Number of treated dental caries ≥ 3 and father’s poor BHPS (0–1) were found to be associated with dental neglect in the univariate analysis; however, these associations were not observed in the multivariate analysis.

**Table 2:** Logistic regression analysis children with dental neglect (*n* = 1655)

	Univariate	Multivariate
	OR (95% CI)	OR (95% CI)
Sex		
Girls	1	1
Boys	1.07(0.62–1.85)	1.00(0.57–1.76)
Grade		1
Low (1st–3rd)	1	1
High (4th–6th)	2.18(1.21–3.91)**	2.08(1.14–3.78)*
Skipping breakfast		
No	1	1
Yes	2.26(1.00–5.14)	1.86(0.79–4.39)
Eating alone at dinner		
No	1	1
Yes	3.45(0.78–15.27)	3.36(0.70–16.03)
Number of treated teeth		
≤ 2	1	1
≥ 3	1.84(1.06–3.19)*	1.68(0.95–2.94)
Father’s BHPS		
Not poor (2–7)	1	1
Poor (0–1)	2.53(1.05–6.10)*	1.73(0.34–8.86)
Mother’s BHPS		
Not poor (2–7)	1	1
Poor (0–1)	3.27(0.74–14.40)	2.04(0.77–5.43)
Father’s Internet and game use at home, h/day		
< 2 h	1	1
≥ 2 h	2.20(1.17–4.11)*	1.99(1.02–3.88)*
Mother’s Internet and game use at home, h/day		
< 2 h	1	1
≥ 2 h	1.46(0.51–4.13)	0.65(0.21–2.02)
Perceived family affluence		
Affluent	1	1
Neither	2.13(0.92–4.94)	1.87(0.79–4.42)
No	3.19(1.34–7.63)**	2.78(1.14–6.81)*
Mother’s employment status		
Full-time	1	1
Part-time	0.78(0.42–1.45)	0.73(0.39–1.38)
Unemployment	1.55(0.75–3.18)	1.78(0.83–3.80)
Number of after school activities		
≥ 1	1	1
0	2.58(1.46–4.55)**	1.99(1.10–3.62)*

Model was adjusted for sex, age, lifestyle, SES

*OR* odds ratio, *CI* confidence interval, significance level, *BPPS* Breslow’s health practice score

*P < 0.05, **P < 0.01, Hosmer-Lemeshow: *P* = 0.38

In the multiple logistic regression analysis that contained all the factors of children’s sex and age, parental lifestyle, and SES associated with dental neglect, the results were 4–6 graders (OR, 2.08; 95% CI, 1.14–3.78), father’s Internet and game usage time for ≥ 2h/day (OR, 1.99; 95% CI, 1.02–3.88), no affluent (OR, 2.78; 95% CI, 1.14–6.81), and number of after school activities = 0 (OR, 1.99; 95% CI, 1.10–3.62).

## Discussion

This study showed that 3.2% of children experienced dental neglect (i.e., had untreated caries despite school dentists recommending treatment). Further, being 4–6 graders, having low SES (i.e., no family affluence or afterschool activities), and father’s internet and game use at home on weekday ≥ 2 h/day were associated with dental neglect.

### Associations of SES with dental neglect

We demonstrated that children with dental neglect were more commonly from non-affluent families than affluent families, even after adjusting for sex, grade, and lifestyle factors of children and their parents. The findings in this study support previous studies that showed associations between SES and dental neglect.

In previous studies, low SES was associated with non-healthy lifestyles [20, 21]. Further, child poverty directly led to maltreatment, particularly neglect, due to a decreasing parental capacity to meet children’s basic needs [21].

In this study, children having no afterschool activities, which we used as a proxy measure of SES, was associated with dental neglect. In this context, it is worth noting that afterschool activities are a lifestyle factor for children. However, this may be influenced by parental interests in educational activities outside school and their ability to afford such activities [28–30].

Furthermore, multiple barriers to accessing dental care may exist among low SES families. In Toyama Prefecture, the National Health Insurance system and subsidiaries from the local government cover all medical expenses for children up to junior high school. Therefore, the issue of dental neglect may be mainly attributable to lack of interest, knowledge, and time to take care of children rather than lack of money in low SES families.

Moreover, untreated dental caries can lead to morbidity, poor quality of life, low self-esteem, and other health problems. Future research should explore what barriers prevent low SES families from taking their children to see a dentist.

### Associations of age and sex with dental neglect

In this study, older age was associated with dental neglect, while the prevalence of dental neglect did not differ between boys and girls. A previous study also showed that children have more untreated dental caries as they get older [1]. There may be several reasons for this. First, children and their parents may consider that deciduous teeth will be replaced with permanent teeth relatively soon. Second, as age increases, children start making their own judgements, and their parents may fail to persuade them. Third, older children have less time to see a dentist because they are busier after school than younger children [32]. Therefore, medical staff may need to explain the importance of going to a dental clinic.

### Associations of lifestyle factors of children with dental neglect

Previous studies reported that skipping breakfast and eating alone at dinner were associated with untreated dental caries and neglect [20–22]. Therefore, in this study, these risk factors were adjusted for in the multivariate analysis. However, such lifestyle factors were not significantly related to dental neglect, although children were likely to have a higher odds ratio of skipping breakfast and eating dinner in terms of dental neglect. This could be because the number of children skipping breakfast and eating dinner alone was small. Other reasons could be that socioeconomic and family factors may be the more important determinants of dental neglect (i.e., the management of dental caries of children in a family) than the lifestyle factors of the children.

### Associations of lifestyle factors of parents with dental neglect

It has been reported that parental lifestyle factors such as skipping breakfast, smoking, alcoholism, drug addiction, low self-esteem, anxiety, high stress, depression, and awareness of a child’s health are associated with children’s untreated dental caries [8, 33–35]. In this study, we also found that children of mothers with undesirable lifestyle factors (i.e., skipping breakfast, snacking, alcohol consumption, short sleep duration, long hours of internet use) were more likely to have dental neglect. Additionally, we found that father’s Internet and game usage time ≥ 2 h is associated with dental neglect as well.

Moreover, a 2018 white paper from Japan’s Ministry of Internal Affairs and Communications reported that TV viewing time is decreasing yearly and internet time is increasing [36]. Television viewing is often carried out with family members, while internet usage is typically carried out individually. A previous study reported that father’s internet usage time ≥ 2 h/day was significantly associated with prolonged screen time for children [12]. In such a home, communication among family members is difficult. Consequently, there may be few opportunities to discuss the health of children within the family. In this context, the findings from this study suggest that parental factors affect not only the development of dental caries in children but also their management.

It has been shown that pathological gaming (TV game, internet game) for mothers and fathers was related to their depression, decreased feeling of parental efficacy, competence, increased stress, and increased impact of parenting [37]. Thus, extended media usage may influence children’s health. Further, parents lacking desirable lifestyles and health knowledge may not take their children to dental clinics. Thus, health education may be needed not only for children but also for parents to improve their understanding of their children’s health. In addition, health professionals should recognize that prolonged parental internet use could be a modern risk factor for the negligence of childcare, as traditional factors such as smoking and alcohol drinking have been [8].

### Strengths and limitations

One of the strengths of this study is that it is a rare research report dealing with dental neglect among a large pool of participants. In addition, this study comprehensively analyzed the associations between SES, lifestyle factors of parents, and lifestyle factors of children and dental caries.

This study has several limitations. First, the lifestyle factors of the parents, those of the children, and the past history of dental caries were self-reported. Second, we were not permitted to ask about household income.

## Conclusions

Low SES and certain parental lifestyle factors were associated with dental neglect in elementary school children. The reasons why these are potential risk factors for dental neglect require further research.

## Data Availability

The datasets analyzed during the current study are available from the corresponding author on reasonable request.

## Abbreviations

CI: Confidence interval
OR: Odds ratio
SES: Socioeconomic status
